# In Pursuit of the Perfect Dancer’s Ballet Foot. The Footprint, Stabilometric, Pedobarographic Parameters of Professional Ballet Dancers

**DOI:** 10.3390/biology10050435

**Published:** 2021-05-14

**Authors:** Joanna Gorwa, Robert Michnik, Katarzyna Nowakowska-Lipiec

**Affiliations:** 1 Department of Biomechanics, Faculty of Sport Sciences, Poznan University of Physical Education, 61-871 Poznań, Poland; 2 Department of Biomechatronics, Faculty of Biomedical Engineering, Silesian University of Technology, 41-800 Zabrze, Poland; Robert.Michnik@polsl.pl (R.M.); Katarzyna.Nowakowska-Lipiec@polsl.pl (K.N.-L.)

**Keywords:** ballet dancers, footprint parameters, Clarke angle, Weissflog index, stabilography

## Abstract

**Simple Summary:**

The purpose of this research was to assess footprint parameters in a group of ballet dancers and the correlation between these parameters with lateralisation, stabilometric parameters, pedo-barographic parameters and work environment. The research was carried out on a group of 44 elite professional ballet dancers—the reference group was 44 students. The results of the test of body balance, thrust under feet and footprint parameters (e.g., Clarke angle and Weissflog index) were analyzed. Statistically significant differences between the groups were observed in relation to the stabilometric parameters, the percentage pressure of the left forefoot and the right heel and the value of the Clarke angle. The obtained results imply that the high arch of the foot is, most probably, inborn and can be increased only slightly through exercise. The occurrence of pes cavus and flatfoot in ballet dancers is not connected to the total and professional career duration and weekly training volume. Practical conclusions drawn from the tests suggest that, during recruitment to ballet schools, it is necessary to pay attention to whether the applicant’s feet are properly arched. It is not recommended for children with pes cavus to practice en pointe.

**Abstract:**

This work aims to assess footprint parameters in a group of professional ballet dancers and to determine the correlation between the aforementioned parameters and lateralization, stabilometric parameters, pedobarographic parameters and work environment conditions. A group subjected to tests consisted of 44 elite professional ballet dancers and the reference group was composed of 44 students. The test of balance and thrust under feet involved 30 s-long free standing with open eyes on a podographic platform. The research-related analysis was concerned with footprint parameters (foot length and width, Clarke angle, and Weissflog index), stabilometric parameters (path length and ellipse field, mean value of the velocity and deflection of the displacement of the center of the foot pressure on the ground) and pedobarographic parameters (percentage thrust on the right, left foot as well as the front and rear part the foot). Statistically significant differences between the groups were observed in relation to the stabilometric parameters, the percentage pressure of the left forefoot and the right heel, as well as the value of the Clarke angle (*p* ≤ 0.05). The longitudinal arch of the foot and the width of the foot in ballet dancers are not dependent on the total and professional career duration and weekly training volume

## 1. Introduction

In the world of professional dance, there is a notion of the “perfect foot”. The perfect foot is every artist’s dream and a door-opener to many elite ballet schools. The phenomenon of the perfect foot results directly from the fact that the classical dance requires very high efficiency of the ankle. The dancer’s foot must perform the maximum dorsal flexion and the maximum plantar flexion, above the maximum ranges in the ankle in relation to the population mean, which ranges between 20° dorsiflexion and 50° plantarflexion [1,2]. The feet of professional ballet dancers may reach a mean ankle-foot plantarflexion of 113° [1]. General suppleness should be accompanied by an appropriate foot arch. A significantly arched foot with a high longitudinal arch guarantees high aesthetics in ballet. According to Simmel [3], the ideally arched foot is accompanied by the vertical position of the tibial bone, tarsal bone, instep and the front part of the foot in an optimal gravity force line, enabling the axial loading of the foot bones, which, from a biomechanical point of view, provides maximum stability when standing en pointé.

However, the question remains whether a significantly arched foot, in spite of its undoubtedly high aesthetic advantages, is capable of meeting technical requirements imposed by classical dance.

Both the ankle and foot itself of professional dancers are exposed to high stresses and loads connected to dance techniques [4,5,6]. Related investigations indicate that injuries affecting these structures constitute between 4.7% and 54% of all injuries suffered by ballet dancers [4,7,8,9]. The most common injuries are ligament abnormalities, impingement syndromes, tendon abnormalities and stress injuries [1].

Most injuries of the ankle are connected with performing “pointé” and “demi pointé” [1,2]. Apart from “turnout”, these two are the most characteristic technical elements of the classical dance [10,11]. Both “pointé” and “demi pointé” are preceded by multi-annual exercises. As Weiss et al. [12] state, muscles responsible for the foot and the ankle must represent an appropriately high level of strength and flexibility before a dancer can start training “toe ascent” (rising on the tips of the toes). According to Liederbach, when performing the above-named element, the dancer generates force equivalent to the 10-fold value of the BW (body weight) [13]. The “ascent” on the top of points requires the work of lower limb muscles which, at the same time, must be both very strong and flexible [14]. Many authors agree that the introduction of the aforesaid exercises before the foot reaches its full functional maturity increases its susceptibility to injuries in the future [12,15]. Solomon (1993) estimated that a plantar flexion of 180° in relation to the central axis of the tibial bone is a condition of the proper, full and safe “en pointé” [2,15]. It is also known that, depending on the type of performance they are preparing for, classical dancers work “on toes” for more than a half of their working day [16].

For dancers, their feet are undoubtedly an important “link” in the mechanism of proper shock absorption [14]. Feet partly absorb impact forces [17] and co-adjust postural and stabilometric parameters [18]. In spite of such a biomechanically important role of this part of the locomotor system, available subject-related reference publications do not contain the analysis of footprint parameters such as the Clarke angle (CI) or the Weissflog index (WI) in relation to ballet dancers.

These two parameters identifying longitudinal and transverse flat feet are essential in dance. They decide the aesthetics desired in ballet (slenderness of the foot and its arches). However, incorrect values of these can be an obstacle in the technical tasks of the dance. The blind pursuit of an aesthetically perfect foot means that, during enrollment in ballet schools, candidates who have a very high Clarke angle value and/or tend to transverse flat feet are accepted. An excessively arched foot (pes calvus) will lose its flexibility over time; it becomes hard and stiff, and its load-bearing capacity drops drastically [3]. Pes calvus is characterized by a significantly reduced contact surface with the ground and this may cause excessive accumulation of loads in this structure and susceptibility to injuries [1]. However, feet with transverse flatfoot, which is defined by the WI index, will exacerbate this defect in the everyday technical tasks of classical ballet [1,2].

This work aimed to assess footprint parameters in a group of ballet dancers and the correlation between these parameters with lateralisation, stabilometric parameters, pedobarographic parameters and work environment conditions.

## 2. Materials and Methods

### 2.1. Participants

The study included 44 elite professional ballet dancers (BD, 25 ± 6 years, 1.75 ± 0.10 m, 62.55 ± 10.22 kg): 22 males (BD_M, 25 ± 5 years, 1.82 ± 0.08, 70.77 ± 6.73 kg) and 22 females (BD_F, 26 ± 6 years, 1.69 ± 0.06 m, 54.32 ± 5.20 kg) working on a full-time basis in two Polish national theatres. They were apparently healthy and did not suffer from any chronic diseases. In the last 6 months before the examination, they had no ankle or foot contusions. All participating artists had danced for at least 8 years (since starting to learn) and graduated from general education schools of ballet. The male and female dancers did not differ as regards age, total and professional career duration, as well as weekly training volume. The check group was composed of students (S, 22 ± 3 years, 1.74 ± 0.09 m, 68.36 ± 12.38 kg): 22 males (S_M, 22 ± 4 years, 1.81 ± 0.06 m, 76.73 ± 11.23 kg) and 22 females (S_F, 22 ± 3 years, 1.67 ± 0.06 m, 60.00 ± 6.40 kg) performing moderate physical activities and studying at the University of Medical Sciences. The male and female students did not differ as regards age. The basic characteristics of the dancers and students are shown in Table 1 and Table 2—the characteristics without division and with division into sexes. The group of the ballet dancers and that of the students, without division into sex, did not differ in regards to body height.

Body mass [kg] and height [cm] were measured to two decimal places by trained personnel according to standardized procedures using a Seca 285 digital measuring station (Seca, Hamburg, Germany). Participants wore only underwear and were without shoes. The BMI was calculated as body weight [kg] divided by height squared [m^2^]. The total career duration was defined as the years between starting ballet school education and the day of examination. The professional career duration was the period (years in decimal notation) between the date of starting occupational activity in a ballet group and the day of examination.

### 2.2. Measurement Protocol

All the test participants agreed to take part in the tests. Measurements in the group of students and that of the dancers were performed by the same person, in the same conditions and using the same equipment. The study design was approved by the Bioethical Committee at the Poznań University of Medical Sciences (Poland) before commencement of the study (decision no. 796/09).

### 2.3. Pedobarography

This study was carried out with the use of a pedobarographic measuring system. The system consisted of a PEL 38 multipoint 1024-sensor plantar-force transducer platform and a Twin 99 software programme (Medicapteurs, Toulouse, France). The platform dimensions were 515 mm × 445 mm and dimensions of an active area were 320 mm × 320 mm (1024 sensors, sensor dimensions 10 mm × 10 mm). Before the tests, the platform reliability was assessed by measuring the pressure from the weight of a model block for the entire platform surface. Then intraclass correlation coefficients were calculated. The consistency of the measurements was 99.79 and the agreement was 96.12. The sampling frequency for measuring was 1000 Hz; i.s. was the maximum sampling frequency offered by the system.

### 2.4. Measuring Plantar Pressure Distribution and Balance Parameters

The test of balance and thrust under feet involved a position of 30 s-long free standing with open eyes. During the test, each person stood freely on two lower limbs set apart at the pelvis width; the upper limbs were placed along the body.

### 2.5. Analysed Parameters

#### 2.5.1. Footprint Parameters

The foot length (L) and width (W), as well as the Clarke angle (CL), were calculated using the Twin 99 software programme, in accordance with instructions by Pauk et al. 2017 [19].

The Clarke angle was determined between the straight line running along the medial edge of the foot and the straight line connecting the point of the deepest indentation of the foot and the point of contact between the medial tangent with the edge of the foot [19]. The Weissflog index (WI) was calculated as the quotient of the foot length (H) and the foot width (W) [20]. All the footprint parameters were measured three times in relation to each limb, and were always by the same person. The result was the arithmetic mean of the measurements.

##### Criteria of Foot Classification

The Clarke angle enables the assessment of the longitudinal arch of the foot. Feet having a CL angle value of less than 42° were classified as the feet with the fallen arch, i.e., the flatfoot (pes planus), feet classified as having a proper (normal) arch were characterised by the CL restricted within the range of 42–54° (pes rectus), whereas feet having CL ≥ 55° were classified as feet with a high arch (pes cavus) [20,21]. The Weissflog index is used to determine transverse flat feet. WI values restricted within the range of 2.0 to 2.5 were classified as representing flat feet, whereas those restricted within the range of 2.5 to 3.0 were values representing normal feet [20].

#### 2.5.2. Stabilometric Parameters

Stabilometric parameters were defined by:

- path length (PL) [mm]—the total path length covered by the COP (distance covered by the foot centre of pressure on the ground during a measurement),
- ellipse area (EA) [mm^2^]—the area of ellipse where the COP was located during a test (area of an ellipsis formed by 95% of COP locations during a test),
- average speed of the displacement of the CoP in transverse axis Xs and sagittal axis Ys, their standard deviations of speed (Xdev, Ydev) and the mean value of speed CoP (AVGQs).

#### 2.5.3. Pedobarographic Parameters

Pedobarographic parameters included the percentage thrust on the right foot (TR—thrust on the right foot) and the left foot (TL—thrust on the left foot), as well as the percentage thrust on the right forefoot and right backfoot (TRF—thrust on the right forefoot, TRB—thrust on the right backfoot) the percentage thrust on the left forefoot and left backfoot (TLF—thrust on the left forefoot, TLB—thrust on the left backfoot).

#### 2.5.4. Work Environment Conditions

Work environment conditions included the total career duration (TC) (years), professional career duration (PC) (years) and weekly training (WT) (hours).

### 2.6. Statistical Analysis of Results

The results obtained in the tests were subjected to statistical analysis. The quantitative variables of the parameters subjected to analysis were described using the mean value, standard deviation, as well as the minimum and maximum value. The normality of the distribution of the analysed variables was verified using the Shapiro-Wilk test. The identification of the relationship between the analysed variables involved the determination of the Spearman rank correlation coefficient or the Pearson linear correlation coefficient in relation to the normality of the distribution of given parameters.

The presence of differences between the analysed parameters in the groups (in relation to sex or between the group of the dancers and that of the students) was verified by performing the Student’s *t*-test for independent samples or the Mann-Whitney U test—depending on the normality of the distribution of analysed variables. The research also involved the performance of the ANOVA GLM factor analysis, verifying whether the selected factors or the interactions of selected factors differentiate stabilometric parameters, the thrust of the forefoot on the ground and the value of the Clarke angle. The level of significance adopted in the statistical analyses was *p* = 0.05. Calculations were performed using a Dell Statistica 13.1 software programme (Dell Inc., Tulusa, Oklahoma, USA) and a PQStat 1.8 software programme (PQStat Software, Poznań, Poland).

## 3. Results

Table 3 presents obtained values of parameters indicating the balance, the manner in which feet are loaded and footprint parameters in the group of the ballet dancers and that of the students in relation to the sex of the test participants.

The Student’s *t*-test or the Mann-Whitney U-test concerning the independent variables revealed that, in relation to nearly all analyzed parameters, there were no statistically significant differences in the group of the dancers and that of the students in relation to sex. Due to the foregoing, at the subsequent stages, statistical analyses were performed without division into sex. Statistically significant differences were only observed in relation to the width and the length of the right and left foot (Table 3). Table 4 presents the values of the analyzed parameters in relation to the group of the ballet dancers and that of the students, regardless of sex.

It was demonstrated that the stabilometric parameters (PL, EA, Xs, Ys, Xdev, and AVGOs) were statistically significantly different in relation to the group of the dancers and that of the students; however, the power of the tests was not high, power = 0.5–0.8, and even very low in relation to EA, i.e., power = 0.05).

Statistically significant differences were recorded in relation to the percentage level of the thrust on the left forefoot (TLF, *p* = 0.007, effect size = 1.91, power = 0.78) and the thrust on the right backfoot (TRB *p* = 0.004, effect size = 1.93, power = 0.82) as well as in relation to the values of the Clarke angle for the right foot (*p* ≤ 0.05, effect size = 6.71, power = 0.99) and the left foot (*p* ≤ 0.05, effect size = 6.2, power = 0.99).

The feet of the dancers and those of the students did not differ in terms of width (W), length (L) and the value of the WI, indicating transverse flat feet (Table 4).

The analysis of the CL values of 88 tested dancers’ feet, revealed 3 cases (3.41%) of a pathological longitudinal arch, 5 cases (5.68%) of a fallen arch, 72 cases (81.82%) of the proper arch and 8 cases (9.09%) of a high arch. The analysis of the CL values of the students’ feet revealed 3 cases (3.41%) of a pathological longitudinal arch, 35 cases (39.77%) of a fallen arch, 49 cases (55.69%) of the proper arch and only one case (1.13%) of a high arch.

The value of the Weissflog index indicating transverse flat feet revealed 38 cases (43.18%) of flat feet and 50 cases (56.82%) of the normal foot in the group of the dancers. In the group of students, there were 28 cases (31.81%) of flat feet and 60 cases (68.19%) of normal feet.

Another stage of the analyses involved the verification of whether stabilometric parameters were conditioned by footprint parameters.

To this end, it was necessary to determine whether the Spearman rank correlation coefficient (because of the lacking normality of the distribution of parameters subjected to analysis) between CL, W and WI in relation to the right and left lower limb and LE and EA separately in relation to the group of the dancers and that of the students (Table 5). It was revealed that, in the group of BD, there were no correlations between stabilometric parameters and footprint parameters. In turn, in the group of the students it was possible to notice statistically significant, yet low, correlations between the Clarke angle and the Weissflog index and the path length, as well as between the Weissflog index and the area of ellipse.

In addition, the statistical analyses revealed that the longitudinal arch of the foot (CL) and foot width (W) did not depend on the dancers’ work environment conditions, i.e., TC, PC and WT (Table 6).

**Figure 1:**
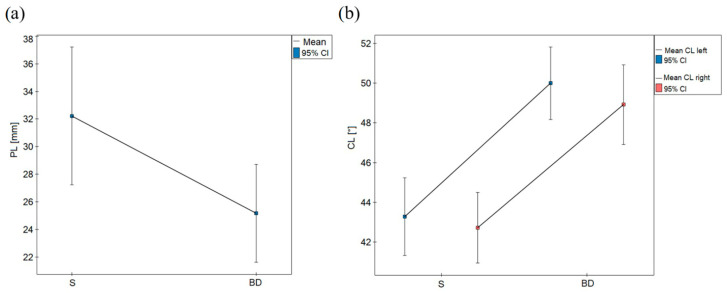
Distribution of values: (**a**) path length (PL) and (**b**) Clarke angle (CL) in relation to the group of students (S) and ballet dancers (BD).

The performed ANOVA GLM factor analysis revealed that the values of stabilometric parameters and the longitudinal arch of the foot (CL) in the group of the students and that of the dancers were not differentiated by such factors as sex, lateralisation and the Weissflog index or interactions of these factors belonging to the group of BD or that of S (Table 7). Only belonging to the group (BD, S) significantly differentiated the statistical values of PL, where lower values were observed in relation to the dancers (Figure 1a). Belonging to a given group explains the variability of PL values in relation to approximately 6% (Eta-square = 0.059).

Belonging to the group (BD, S) statistically significantly differentiated the values of the Clarke angle—higher values of CL were obtained in relation to the dancers (Figure 1b). Belonging to a group explains the variability of CL in relation to the right foot in approximately 20% (Eta-square = 0.201) and in relation to the left foot in approximately 23% (Eta-square = 0.229).

## 4. Discussion

The primary findings are as follows: (1) professional dancers are characterized by the statistically significantly higher longitudinal arch of the foot, identified as the Clarke angle value and significantly better stabilometric parameters (CoP path length, the area of ellipse, speed in direction X and Y) than the check group, (2) there is a statistically significant correlation between footprint parameters and stabilometric parameters only in the check group; there is no such correlation in the group of professional dancers, (3) in the group of the professional artists, footprint parameters (the Clarke angle, the Weissflog index and the width of the foot) are not connected with work environment conditions such as the total career, professional career and weekly training, and (4) transverse flat feet defined as a WI value of above 2.5 was observed in as many as 19 (21%) professional dancers.

Presented below are certain possible explanations, interpretations and suggestions based on the data obtained in the tests.

### 4.1. Selection of Ballet Schools and Clarke Angle

The CL results obtained in the article indicate that 81.82% of ballet dancers’ feet have a proper arch, and 9% of feet exhibit high arches. While in the reference group proper arches were recorded in less than 40% of cases, only for one person did the results of CL indicate a foot with a high arch. The remaining values indicate the presence of a pathological longitudinal arch (flat foot).

It should be emphasized that the statistical analyses carried out in the study showed that the state of the longitudinal arch of the foot (CL angle) does not depend on the conditions of the dancers’ work environment (TC, PC, and WT), i.e., it probably does not change during their professional career. Interestingly, the ANOVA GLM factor analysis performed showed that belonging to a group of dancers or students explains the value of CL in over 20%.

The height of the longitudinal arch of the foot seems to be an inborn feature [22]. Ballet exercises may slightly increase the aforementioned feature, yet they primarily affect foot muscles [23].

The foregoing was confirmed by the results of the test performed by the authors. The so-called high arch does not depend on the total career duration, weekly training volume or professional career duration of the dancers. The authors suppose that high values of the Clarke angle recorded in relation to the group of dancers most likely result from the selection to ballet schools and hereditary features rather than ballet practice and exercises.

During the above-mentioned selection of candidates, a wish to find the owners of such a high arch becomes a priority and leads to the selection of dancers. Unfortunately, this type of foot later becomes incapable of satisfying enormous technical requirements of the classical dance [3]. Pes cavus stands for an excessively arched foot (high-arch foot). During the tests, the aforesaid type of the foot was found in 4 dancers (9.1%). With time, pes cavus loses its flexibility, becomes hard and stiff, and its ability to transmit loads falls dramatically [3]. The lack of flexibility reduces shock-absorbing properties in the foot, potentially leading to sprained ankles, overloads and instep fractures [1]. For this reason, during the recruitment to ballet schools, it is of utmost importance to be able to distinguish the high arch (desirable in terms of dance), restricted within the Clark angle range of 48 degree to 55 degree from pes cavus > 55 degree [3]. The excessively arched foot is characterised by a significantly smaller area of contact with the ground. The afore-mentioned foot leads to the excessive accumulation of loads responsible for strong pain accompanying the career of professional dancers [1].

### 4.2. Foot Mechanics and Clarke Angle

The statistical analyses carried out in this study showed that the value of the Clarke angle is significantly different in the group of dancers and students (*p* ≤ 0.05). The mean values of the Clarke angle are much higher in the group of dancers. In the group of students, the mean Clarke angles were 43.28° ± 6.43° for the left foot and 42.72° ± 5.83° for the right foot, while in the group of dancers: 49.99° ± 6.00° for the left and 48.92° ± 6.61° for the right foot. Higher Clarke values mean that dancers likely have shortened plantar fascia, and this may result in higher values of the forces generated by the posterior calf muscles.

In terms of mechanics, the type of lever in the ankle during dance depends on how forces are applied to the foot and the location of the point of support. When making relevé, the angle is the second-class lever. In the second-class lever, resistance (body weight) is located between the point of support and applied force. Then, the point of support is the metatarsophalangeal area on the floor, resistance is the body weight affecting the ankle and the action force is exerted by the calf muscle and the soleus muscle acting through the Achilles tendon. “Standing” en pointé requires the isometric contraction of the muscles combined with the simultaneous maintaining of the centre of gravity over a relatively small area of support located on the tips of the toes [2].

Deforth et al. [24] demonstrated that the “scooped” foot with the high arch (Clarke angle > 55 deg) is characterised by the shorter arm of the force transmitted by the Achilles tendon. Because of this, during shock absorption after landing, precisely discussed by Gorwa et al. [14], in relation to such a highly arched foot, muscular force generated by the muscles involved in shock absorption must be proportionally higher (to the shorter arm of the force). A similar muscular work will accompany the ascent to relevé. The shorter arm of the force will require greater involvement of the calf muscle. The fact that variously arched feet affect the landing phase after a jump was discussed by Juhyun Kim and Kyungock Yi [25]. According to the authors, the extensive impulse during landing according to a foot type was significantly larger in the case of flexible pes planus than pes rectus. Regrettably, there are no tests concerning pes calvus and its shock absorbability.

In addition, a very high arch may shorten plantar aponeurosis. The dysfunction of the aforesaid structure may trigger a sequence of events and affect the Achilles tendon, calf muscles, the back part of the thigh (hamstrings), and even the lower parts of the spine. According to the latest research [26], force generation on the hallux is more affected by the ankle joint angle than the lesser toes. A similar situation can be observed as regards the hallux valgus, related to the deformation of the spine, positions of the lower limbs and the range of movements in the ankle [27].

### 4.3. Stabilometric and Pedobarographic Parameters and Learning the Ballet Technique

The research has shown that the load distributions between the right and left limbs are even (approx. 50% each) both in the group of dancers and in the reference group. Statistically significant differences between the group of dancers and students were noted for the percent level of thrust on the left forefoot (TLF, *p* = 0.007) and the right heel (TRB, *p* ≤ 0.05).

The stabilometric parameters differ statistically between the group of dancers and students, i.e., path length (*p* = 0.015), elipse area (*p* = 0.036), mean value of speed CoP (*p* = 0.038), average speed of the displacement of the CoP in the transverse axis (*p* = 0.017), average speed of the displacement of the CoP in sagittal axis (*p* = 0.047) and standard deviations of Xs speed (*p* = 0.004).

This indicates a much better ability of the dancers to maintain balance, which is the result of the dancers’ work, i.e., constant balance exercise. Much lower values of the CoP velocity during the test may indicate a much better body control.

The technique of classical dance is very demanding as regards the foot and often requires a non-physiological posture of the entire body [2]. In ballet schools, in forms 1–3 (children of 10–12 years old), children learn each pas [28]. A significant emphasis is given to an appropriate level of muscular force and feet flexibility. However, the assessment of the above-named parameters is very subjective and not supported by research performed on a regular basis. The second year of education involves the introduction of demi pointe exercises with the pole. Exercises in this position trigger the transfer of load on the heads of instep bones, where the foot subjected to unnatural loads and adopts an unnatural position [2,12]. Children’s bones, being still at the ossification stage, are then particularly susceptible to deformations [12]. It was demonstrated that specific movement tasks of classical dance affect the dancer’s locomotor system structure. The vertical components of ground reaction forces act locally on the ballet dancer’s skeletal system making femoral necks highly mineralised and radial bones (not loaded) osteopenic [29]. The adoption of ballet positions is responsible for the unnaturally high involvement of lower limb muscles and pathological pelvis positions [23]. At the same time, feet exercises lead to specific geometric changes in the aforesaid structures, such as hallux valgus [27].

In dance education, exercises increasing the muscular force of feet are introduced in forms 4–5 (at the age of 13 and 14) [28]. The aforesaid force must be sufficiently high to overcome the ground reaction force of grand jumps (grand jete, sissonne ouverte and grand pas de chat) generating a GRF of 4–9 BW [13,14] and responsible for difficult work in points [12]. In forms 7–9, the dance curriculum, in addition to grand jumps, includes pirouettes and intense work in points, introduced to enable the performance of grand fouette en tournant, cabriole or entrechat-six [28].

Demi pointe and en pointé dance is responsible for the excessive load of the forefoot and toes [2], whereas the forcing of turnout, i.e., the “turning” of ballet positions from the level of feet is responsible for the significant loading of foot muscles [10,23]. Very frequently, it is possible to notice the widening of the forefoot and the thickening of bone attachments [1,2]. The research-related tests performed by the authors did not reveal any statistically significant differences between the width of the foot in the dancers and the students. In addition, the tests did not reveal any correlation between the professional career duration, total career duration and weekly training volume and the width of the foot in the dancers. However, 19 artists participating in the tests had transversely flat feet. Many authors claim the foregoing to result from demi pointé [1,2], as this exercise is responsible for the loading of the heads of the instep bones, particularly of the second and third toe as well as specific and unnatural positions of ligaments. According to Russell et al. [2] “Upon moving to demi-plié the positions of the ligaments do not change appreciably, but their orientations relative to the fibula change. The anterior talofibular ligament’s angle with the fibula becomes more acute and the calcaneofibular ligament is nearly parallel to the fibula. When en pointé, the anterior talofibular ligament is virtually parallel to the fibula, while the calcaneofibular swings posteriorly to a somewhat horizontal position”. This anatomic-biomechanical unnaturality is responsible for the fact that the foot is loaded otherwise than during regular locomotion [20,30].

Statistically significant differences between the group of the dancers and that of the students were noticed in relation to the percentage thrust on the left forefoot (TLF) and the right backfoot (TRB). It seems that such a strange result could be ascribed to a certain “manner” functioning in the circle of professional dancers. During a standing position, the left lower limb is shifted forward, whereas the right lower limb is moved backwards. This free position, common in the world of dance, is also adopted by dancers of other styles. It is possible that the above-presented habit affects general parameters related to stability and the distribution of forces under feet. However, the aforementioned speculations require further research.

### 4.4. Very High Arch versus Ballet

The high arch in ballet has primarily aesthetic qualities (although its lack may really preclude the performance of exercises and entirely exclude classical dance) [3]. In terms of the technical side of ballet, a very high arch is spectacular during work in the air but rather problematic during work on the floor. For instance, to perform ballonné on the toe the dancer must wear soft “dead” points enabling the standing “on the hook” on the tip. In turn, to stand on arabesqué or to perform a pirouette, the points should be “new”. It is difficult to combine both such features in the same pair of points [31]. Persons with the “inferior” arch exercise in softer points (dead pointe) which facilitate the performance of various elements. Without major problems, such dancers make ballonné and stand on arabesqué in “dead points”. Dancers having significantly arched feet must change points depending on various technical tasks [31].

However, the obsessive pursuit of the higher arch of the foot through exercises at various stages of education is utterly pointless, as neither the number of hours per week spent on training nor the total or professional career duration have any connection with the value of the Clarke angle.

In the check group, there was a statistically significant correlation between footprint parameters and stabilometric parameters. No such correlation was observed in the group of professional dancers. The authors believe that the foregoing could be ascribed to the specific work of dancers who have to maintain balance on various types of the stance plane and control their body posture in a more automated manner [32]. According to calculations performed by the authors, when standing en pointé, the area of support amounts to approximately 15 cm^2^; in spite of this, the dancer performs arabesque at that moment.

Depending on a performance they are preparing for, the classical dancers work “on toes” for more than a half of their working day [16]. The dancers participating in the measurements work on the average 47.8 h per week. This means, that, on average, dancers spend as many as 24 h on points! Feet exposed to such an accumulation of loads in time, in the order of the 10-fold dancer’s body weight (according to Liederbach) are bound to suffer from fatigue-related injuries [13].

### 4.5. Limitations, and the Direction of Further Research

The pes cavus foot can be pronated or supinated and can be a stable or unstable foot independent of the footprint. Therefore, for a full assessment of the real biomechanical behavior of the foot, in addition to footprint parameters, the Foot Posture Index should be included [33]. In future research of ballet dancers, we plan to include the Foot Posture Index, which, in our opinion, along with footprint parameters, should be introduced during selection to dance schools. Moreover, we believe that to investigate the effect of classical dance on the foot, it would be necessary to perform additional longitudinal tests performed at the beginning of the dancer’s education and at the beginning of the dancer’s professional career.

## 5. Conclusions

The test results imply that the Clarke angle in ballet dancers is not connected with the total and professional career duration and weekly training volume. In the group of professional dancers, there was no correlation between the footprint parameters and the stabilometric parameters. At the same time, the results of the stabilographic tests revealed that the dancers were characterised by better coordination-related skills enabling better maintenance of balance. The lack of correlation between the footprint parameters and stabilometric parameters as well as pedobarographic parameters in the group of dancers may result from the fact that these specific professionals perform balance exercises on various types, shapes and surfaces of stance planes.

## Figures and Tables

**Table 1:** Participants’ demographic characteristic by sex.

	BD_M (*n* = 22)	BD_F (*n* = 22)	Difference (*p* Value)	95% CI	S_M (*n* = 22)	S_F (*n* = 22)	Difference (*p* Value)	95% CI
Age (mean ± SD [years])	25 ± 5	26 ± 6	1 (*p* = 0.46)	−2.08 to 4.54	22 ± 4	22 ± 3	−0.82 (*p* = 0.39)	−2.70 to 1.07
Body height (mean ± SD [m])	1.82 ± 0.08	1.69 ± 0.06	−0.14 (*p* ≤ 0.05 *)	−0.18 to −0.09	1.81 ± 0.06	1.67 ± 0.06	−0.13 (*p* ≤ 0.05 *)	−0.17 to −0.10
Body mass (mean ± SD [kg])	70.77 ± 6.73	54.32 ± 5.20	−16.46 (*p* ≤ 0.05 *)	−20.11 to 12.80	76.73 ± 11.23	60.00 ± 6.40	−16.73 (*p* ≤ 0.05 *)	−22.29 to −11.17
BMI (mean ± SD [kg/m^2^])	21.33 ± 1.17	19.12 ± 1.52	−2.21 (*p* ≤ 0.05 *)	−3.03 to −1.38	23.51 ± 2.91	21.44 ± 2.04	−2.07 (*p* = 0.01 *)	−3.60 to −0.55
Lower limb lateralization Right/Left [number]	19/3	17/5	-	-	16/6	22/0	-	-
Professional career (mean ± SD [years]/[months])	6.08 ± 3.40/ 73.00 ± 40.78	6.95 ± 4.49/ 83.38 ± 53.84	0.87 (*p* = 0.47)	−1.56 to 3.29	-	-	-	-
Total career (mean ± SD [years])	14.64 ± 4.05	16.27 ± 5.73	1.63 (*p* = 0.28)	−1.38 to 4.66	-	-	-	-
Weekly training (means ± SD [hours])	46.91 ± 6.73	48.68 ± 6.39	1.77 (*p* = 0.38)	−2.22 to 5.77	-	-	-	-

* *p* ≤ 0.05; BD_M—male ballet dancers, BD_F—female ballet dancers, S_M—male students, S_F—female students, CI—confidence interval.

**Table 2:** Participants’ demographic characteristic without division by sex.

	BD (*n* = 44)	S (*n* = 44)	Difference (*p* Value)	95% CI
Age (mean ± SD [years])	25 ± 6	22 ± 3	−3 (*p* ≤ 0.05 *)	−5.30 to −1.56
Body height (mean ± SD [m])	1.75 ± 0.10	1.74 ± 0.09	−0.01 (*p* = 0.48)	−0.05 to 0.03
Body mass (mean ± SD [kg])	62.55 ± 10.22	68.36 ± 12.38	5.81 (*p* = 0.02 *)	1.01 to 10.63
BMI (mean ± SD [kg/m^2^])	20.22 ± 1.75	22.48 ± 2.69	2.26 (*p* ≤ 0.05 *)	1.29 to 3.22
Lower limb lateralization Right/Left [number]	36/8	38/6	-	-
Professional career (mean ± SD [years]/[months])	6.52 ± 3.96/78.19 ± 47.49	-	-	-
Total career (mean ± SD [years])	15.45 ± 4.97	-	-	-
Weekly training (means ± SD [hours])	47.80 ± 6.55	-	-	-

* *p* ≤ 0.05; BD—ballet dancers, S—students, CI—confidence interval.

**Table 3:** Stabilometric parameters, pedobarographic parameters and footprint parameters in the group of ballet dancers and students with division by sex.

	BD_M		BD_F		BD_M vs. BD_F	S_M		S_F		S_M vs. S_F
	Mean ± SD	Min–Max	Mean ± SD	Min–Max	*p* Value	Mean ± SD	Min–Max	Mean	Min–Max	*p* Value
TLF [%]	24.59 ± 1.53	23–27	23.77 ± 2.78	18–29	0.257	22.41 ± 4.1	10–30	22.14 ± 3.98	15–30	0.824
TRF [%]	27.32 ± 2.77	21–32	27.36 ± 2.75	20–33	0.957	27.27 ± 5.28	18–42	26.45 ± 3.91	20–35	0.991
TLB [%]	24 ± 1.69	21–27	24.55 ± 3.43	20–35	0.749	24.45 ± 6.01	10–40	25.18 ± 4.7	14–34	0.657
TRB [%]	24.05 ± 2.21	18–28	24.27 ± 2.43	17–29	0.638	25.82 ± 4.26	14–32	26.36 ± 3.29	20–32	0.637
TL [%]	48.68 ± 1.52	46–51	48.27 ± 1.96	45–53	0.372	48.40 ± 2.35	44–53	47.09 ± 2.16	42–50	0.292
TR [%]	51.32 ± 1.52	49–54	51.91 ± 1.63	49–55	0.223	51.60 ± 2.35	47–56	52.91 ± 2.16	50–58	0.349
PL [mm]	23 ± 8	12–39	28 ± 14	12–74	0.316	33 ± 14	14–68	32 ± 19	14–95	0.489
EA [mm^2^]	7 ± 3	2–13	18 ± 29	3–135	0.081	14 ± 8	4–35	12 ± 9	4–41	0.122
AVGQs [mm/s]	0.7 ± 0.23	0.4–1.2	0.82 ± 0.41	0.4–2.2	0.347	0.98 ± 0.42	0.4–2	0.94 ± 0.6	0.4–2.9	0.327
Xs [mm/s]	0.5 ± 0.19	0.2–0.9	0.55 ± 0.32	0.2–1.7	0.869	0.68 ± 0.29	0.3–1.6	0.64 ± 0.43	0.2–2.1	0.218
Ys [mm/s]	0.48 ± 0.17	0.3–0.9	0.59 ± 0.29	0.3–1.5	0.217	0.7 ± 0.32	0.2–1.4	0.65 ± 0.46	0.3–2.4	0.251
Xdev [mm/s]	0.63 ± 0.23	0.3–1	0.72 ± 0.36	0.3–1.5	0.606	1.1 ± 0.64	0.4–2.8	0.87 ± 0.5	0.2–2.3	0.199
Ydev [mm/s]	1.8 ± 4.31	0.1–21	1.35 ± 0.78	0.4–3.5	0.083	1.35 ± 0.62	0.5–2.8	1.02 ± 0.58	0–2.3	0.083
CL left [deg]	49.87 ± 7.18	30.43–61.4	50.12 ± 4.7	41.6–57.87	0.553	43.88 ± 6.64	30.03–58.53	42.68 ± 6.31	25.03–53	0.541
CL right [deg]	48.98 ± 8.15	26.5–66.43	48.85 ± 4.8	39.93–54.87	0.676	42.77 ± 6.34	28.5–53.8	42.66 ± 5.42	31.03–51.37	0.952
W left [cm]	10.27 ± 0.74	9.22–11.63	9.47 ± 0.57	8.49–10.51	≤0.05 *	10.08 ± 0.79	8.84–11.81	9.44 ± 0.76	7.72–10.31	0.008 *
W right [cm]	10.46 ± 0.71	9.18–11.71	9.59 ± 0.63	8.3–10.81	≤0.05 *	10.28 ± 0.75	8.89–11.71	9.75 ± 0.67	8.16–10.97	0.017 *
L left [cm]	26.40 ± 0.08	24.81–27.99	23.99 ± 1.00	22.21–26.30	≤0.05 *	26.18 ± 1.38	24.1–29.93	24.23 ± 0.86	22.63–25.63	≤0.05 *
L right [cm]	26.28 ± 1.00	24.82–27.86	23.98 ± 1.00	22.39–26.52	≤0.05 *	26.19 ± 1.38	24.03–29.9	24.29 ± 0.84	22.63–25.73	≤0.05 *
WI left	2.58 ± 0.17	2.31–2.93	2.54 ± 0.13	2.29–2.74	0.464	2.61 ± 0.15	2.16–2.79	2.59 ± 0.2	2.26–3.01	0.265
WI right	2.52 ± 0.14	2.31–2.78	2.51 ± 0.13	2.25–2.76	0.917	2.55 ± 0.15	2.18–2.75	2.5 ± 0.17	2.25–2.99	0.284

* *p* ≤ 0.05; TLF—thrust on the left forefoot, TRF—thrust on the right forefoot, TLB—thrust on the left backfoot, TRB—thrust on the right backfoot, TL—thrust on the left foot, TR—thrust on the right foot, PL—path length, EA—elispse area, AVGQs—mean value of speed CoP, Xs—average speed of the displacement of the CoP in transverse axis, Ys—average speed of the displacement of the CoP in sagittal axis, Xdev—standard deviations of Xs speed, Ydev—standard deviations of Ys speed, CL left/right—Clarke angle for left/right foot, W left/right—left/right foot width, L left/right—left/right foot length, WI left/right—Weissflog index for left/right foot, BD_M—male ballet dancers, BD_F—female ballet dancers, S_M—male students, S_F—female students.

**Table 4:** Stabilometric parameters, pedobarographic parameters and footprint parameters in the group of ballet dancers and students without division by sex.

	BD		S		BD vs. S
	Mean ± SD	Min–Max	Mean ± SD	Min–Max	*p* Value
TLF [%]	24.18 ± 2.25	18–29	22.27 ± 4.00	10–30	0.007 *
TRF [%]	27.34 ± 2.73	20–33	26.86 ± 4.61	18–42	0.257
TLB [%]	24.27 ± 2.69	20–35	24.82 ± 5.34	10–40	0.242
TRB [%]	24.16 ± 2.30	17–29	26.09 ± 3.77	14–32	0.004 *
TL [%]	48.48 ± 1.75	45–53	47.72 ± 2.32	42–53	0.069
TR [%]	51.62 ± 1.59	49–55	52.29 ± 2.32	47–58	0.104
PL [mm]	25 ± 12	12–74	32 ± 16	14–95	0.015 *
EA [mm^2^]	12 ± 21	2–135	13 ± 9	4–41	0.036 *
AVGQs [mm/s]	0.76 ± 0.34	0.4–2.2	0.96 ± 0.51	0.4–2.9	0.038 *
Xs [mm/s]	0.52 ± 0.26	0.2–1.7	0.66 ± 0.36	0.2–2.1	0.017 *
Ys [mm/s]	0.53 ± 0.24	0.3–1.5	0.68 ± 0.39	0.2–2.4	0.047 *
Xdev [mm/s]	0.68 ± 0.30	0.3–1.5	0.99 ± 0.58	0.2–2.8	0.004 *
Ydev [mm/s]	1.58 ± 3.07	0.1–21.0	1.18 ± 0.62	0–2.8	0.694
CL left [deg]	49.99 ± 6.00	30.43–61.40	43.28 ± 6.43	25.04–58.53	≤0.05 *
CL right [deg]	48.92 ± 6.61	26.5–66.43	42.72 ± 5.83	28.5–53.8	≤0.05 *
W left [cm]	9.87 ± 0.77	8.49–11.63	9.76 ± 0.83	7.72–11.81	0.513
W right [cm]	10.02 ± 0.79	8.30–11.71	10.01 ± 0.75	8.16–11.71	0.953
L left [cm]	25.20 ± 1.59	22.63–25.63	25.21 ± 1.51	22.63–29.93	0.970
L right [cm]	25.13 ± 1.53	22.63–25.73	25.24 ± 1.48	22.63–29.9	0.730
WI left	2.56 ± 0.15	2.29–2.93	2.60 ± 0.18	2.16–3.01	0.31
WI right	2.51 ± 0.14	2.25–2.78	2.53 ± 0.16	2.18–2.99	0.644

* *p* ≤ 0.05; TLF—thrust on the left forefoot, TRF—thrust on the right forefoot, TLB—thrust on the left backfoot, TRB—thrust on the right backfoot, TL—thrust on the left foot, TR—thrust on the right foot, PL—path length, EA—elispse area, AVGQs—mean value of speed CoP, Xs—average speed of the displacement of the CoP in transverse axis, Ys—average speed of the displacement of the CoP in sagittal axis, Xdev—standard deviations of Xs speed, Ydev—standard deviations of Ys speed, CL left/right—Clarke angle for left/right foot, W left/right—left/right foot width, L left/right—left/right foot length, WI left/right—Weissflog index for left/right foot, BD—ballet dancers, S—students.

**Table 5:** Correlation between stabilometric parameters and footprint parameters—results of the Spearman rank correlation.

		CL Left & PL	CL Right & PL	W Left & PL	W Right & PL	WI Left & PL	WI Right & PL
S	r	0.32	0.35	−0.19	−0.2	0.39	0.38
	*p*	0.03 *	0.02 *	0.23	0.2	0.01 *	0.01 *
BD	r	−0.11	−0.15	−0.1	−0.1	0.11	0.12
	*p*	0.5	0.33	0.51	0.51	0.5	0.45
		CL left & EA	CL right & EA	W left & EA	W right & EA	WI left & EA	WI right & EA
S	r	0.28	0.2	−0.14	−0.09	0.42	0.36
	*p*	0.07	0.19	0.35	0.55	≤0.05 *	0.02 *
BD	r	−0.08	−0.13	−0.12	−0.17	0.06	0.11
	*p*	0.61	0.42	0.42	0.28	0.71	0.46

* *p* ≤ 0.05; CL left/right—Clarke angle for left/right foot, W left/right—left/right foot width, WI left/right—Weissflog index for left/right foot, PL—path length, EA—elispse area, BD—ballet dancers, S—students.

**Table 6:** Correlation between the longitudinal arch of the foot and work environment conditions—results of the Spearman rank correlation.

	CL Left & TC [years]	CL Right & TC [years]	CL Left & PC [years]	CL Right & PC [years]	CL Left & PC [months]	CL Right & PC [months]	CL Left & WT [hours]	CL Right & WT [hours]
r	−0.19	−0.23	−0.24	−0.20	−0.23	−0.19	−0.11	−0.11
*p*	0.11	0.06	0.12	0.19	0.13	0.20	0.49	0.49
	W left & TC [years]	W right & TC [years]	W left & PC [years]	W right & PC [years]	W left & PC [months]	W right & PC [months]	W left & WT [hours]	W right & WT [hours]
r	−0.16	−0.16	−0.13	−0.12	−0.13	−0.12	0.03	−0.01
*p*	0.29	0.29	0.40	0.44	0.39	0.44	0.86	0.93

* *p* ≤ 0.05; CL left/right—Clarke angle for left/right foot, W left/right—left/right foot width, TC—total career duration, PC—professional career duration, WT—weekly training.

**Table 7:** Results of ANOVA GLM factor analysis.

		Group (BD, S)	Sex	Lateralization	WI Right	WI Left	Group & Sex	Group & Lateralization	Group & WI Right	Group & WI Left
PL	F	5.35	0.53	3.29	3.03	3.43	0.83	-	0.37	0.29
	*p*	0.02 *	0.47	0.13	0.09	0.07	0.36	-	0.55	0.59
EA	F	0.01	1.55	0.01	0.12	0.00	3.40	1.29	1.73	1.94
	*p*	0.92	0.22	0.92	0.73	0.98	0.07	0.26	0.19	0.17
CL right	F	21.26	0.01	0.69	0.10	-	0.00	2.22	0.12	-
	*p*	≤0.05 *	0.93	0.41	0.75	-	0.99	0.14	0.73	-
CL left	F	25.19	0.13	0.45	-	0.93	0.29	2.42	-	0.24
	*p*	≤0.05 *	0.72	0.50	-	0.34	0.59	0.12	-	0.62

* *p* ≤ 0.05; PL—path length, EA—elispse area, CL left/right—Clarke angle for left/right foot, WI left/right—Weissflog index for left/right foot, BD—ballet dancers, S—students.

## Data Availability

The datasets used and/or analyzed during the current study are available from the corresponding author on reasonable request.
